# Redox Modulation of Meniere Disease by *Coriolus versicolor* Treatment, a Nutritional Mushroom Approach with Neuroprotective Potential

**DOI:** 10.2174/1570159X22666231206153936

**Published:** 2023-12-08

**Authors:** Rosanna Di Paola, Rosalba Siracusa, Roberta Fusco, Marialaura Ontario, Gaetano Cammilleri, Licia Pantano, Maria Scuto, Mario Tomasello, Sestina Spanò, Angela Trovato Salinaro, Ali S. Abdelhameed, Vincenzo Ferrantelli, Antonio Arcidiacono, Tilman Fritsch, Gabriella Lupo, Anna Signorile, Luigi Maiolino, Salvatore Cuzzocrea, Vittorio Calabrese

**Affiliations:** 1 Department of Veterinary Science, University of Messina, 98168, Messina, Italy;; 2 Department of Chemical, Biological, Pharmaceutical and Environmental Sciences, University of Messina, Messina, Italy;; 3 Department of Biomedical and Biotechnological Sciences; Department of Medical, Surgical Advanced Technologies “G.F. Ingrassia”, University of Catania, Italy;; 4 Food Department, Istituto Zooprofilattico Sperimentale della Sicilia, via Gino Marinuzzi 390129 Palermo, Italy;; 5 Department of Pharmaceutical Chemistry, College of Pharmacy, King Saud University, Riyadh 11451, Kingdom of Saudi Arabia;; 6 Department of Translational Biomedicine and Neuroscience, University of Bari, Aldo Moro, 70124, Bari, Italy;; 7 NAM Institute, Rochusgasse 13, 5020 Salzburg, Austria

**Keywords:** Redoxomics, meniere’s disease, glutathione, neurodegenerative diseases, *Coriolus versicolor*, vitagene pathway, inflammation

## Abstract

**Background:**

Meniere's disease (MD) is a cochlear neurodegenerative disease. Hearing loss appears to be triggered by oxidative stress in the ganglion neurons of the inner ear.

**Objectives:**

Here, we confirm the variation of markers of oxidative stress and inflammation in patients with Meniere and hypothesize that chronic treatment with Coriolus mushroom helps in the response to oxidative stress and acts on α-synuclein and on NF-kB-mediated inflammatory processes.

**Methods:**

Markers of oxidative stress and inflammation were evaluated in MD patients with or without Coriolus treatment for 3 or 6 months.

**Results:**

MD patients had a small increase in Nrf2, HO-1, γ-GC, Hsp70, Trx and sirtuin-1, which were further increased by Coriolus treatment, especially after 6 months. Increased markers of oxidative damage, such as protein carbonyls, HNE, and ultraweak chemiluminescence, associated with a decrease in plasma GSH/GSSG ratio, were also observed in lymphocytes from MD patients. These parameters were restored to values similar to the baseline in patients treated with Coriolus for both 3 and 6 months. Furthermore, treated MD subjects showed decreased expression of α-synuclein, GFAP and Iba-1 proteins and modulation of the NF-kB pathway, which were impaired in MD patients. These changes were greatest in subjects taking supplements for 6 months.

**Conclusion:**

Our study suggests MD as a model of cochlear neurodegenerative disease for the identification of potent inducers of the Nrf2-vitagene pathway, able to reduce the deleterious consequences associated with neurodegenerative damage, probably by indirectly acting on a-synuclein expression and on inflammatory processes NF-kB-mediated.

## 1 INTRODUCTION

**Fig. (1):**
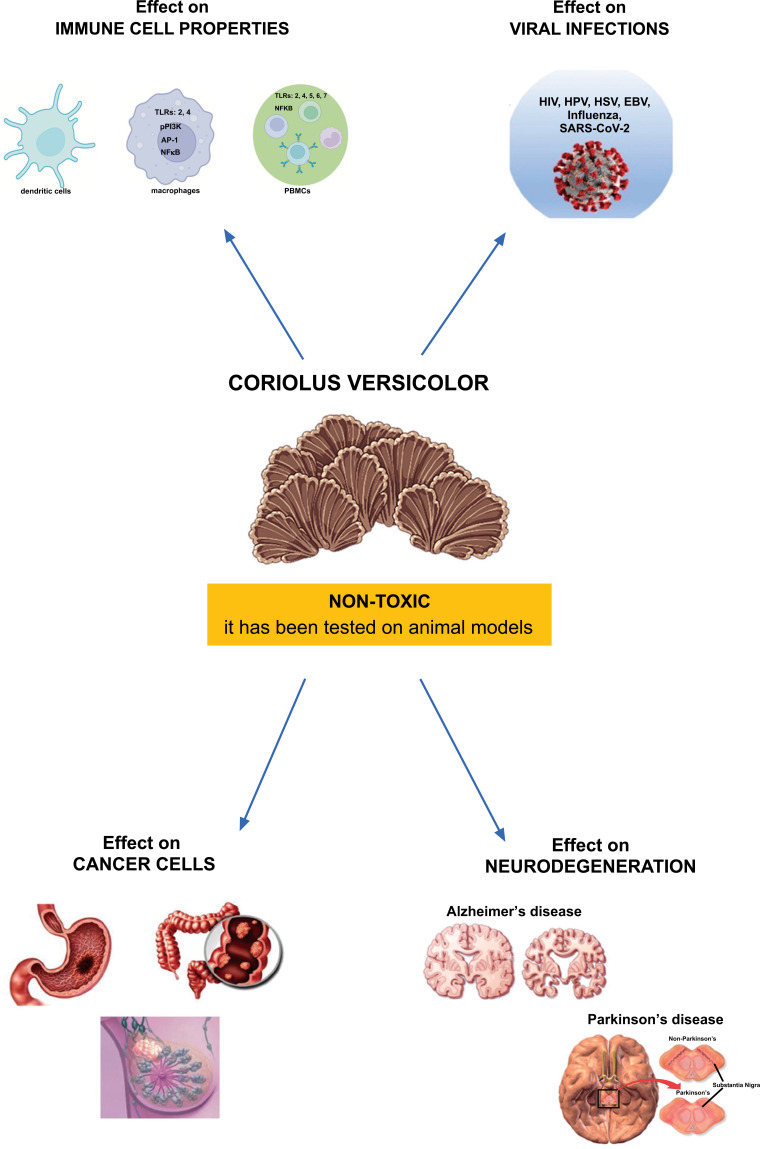
*Coriolus versicolor* properties.

Meniere's disease (MD) is a disabling inner ear syndrome characterized by fluctuating hearing loss, recurrent episodic vertigo, aural fullness, and tinnitus, with nearly one-third of patients completely disabled as a result of the illness, and 7% of these patients suffer from sudden falls named otolithic crises or drop attacks thought to derive from the vestibular otolithic organs, the utricle or saccule. Despite more than an epoch of research since Prosper Meniere's original publication in Manière’s in 1861, the etiology of endolymphatic hydrops and its link to the incapacitating Meniere's bouts of vertigo and hearing loss remains unexplained. In the United States, the occurrence of MD is around 200 cases per 100,000 people [1]. This would imply that the majority of Meniere's patients had the condition for an average of 50 years [2]. Despite new data indicates that oxidative stress and neuroinflammation may play a significant role in the growth of endolymphatic hydrops and subsequent otolithic degeneration, the specific etiology of MD remains uncertain. Several animal and human investigations have also shown that neurosensorial degeneration in spiral ganglion cells correlates with endolymphatic hydrops [3, 4]. As a result, patients' quality of life can suffer significantly as a result of reduced social relationships, physical activity, increased fatigue, diminished work capacity, and, ultimately, neurological and psychiatric disorders such as anxiety or, in the case of intractable MD, neuroses and/or depression [5-8]. Except for symptomatic treatments that signal the need for innovative medical procedures, no particular medical or surgical treatment that might convincingly alter the long-term course of the disease is available to date. In this perspective, the preservation of hearing loss using natural medication candidates has gained significance in recent years as a preventative and therapeutic objective [9, 10]. Mushroom supplementation is a new dietary strategy for reducing oxidative stress by upregulating Nrf2-dependent antioxidant pathways [5-11]. Emerging data suggests that Nrf2-regulated vitagenes are proteins capable of increasing stress resilience pathways in a wide range of human pathologies, including cancer, metabolic, and neurological illnesses [12-16]. As a result, stimulation of Nrf2-dependent resilience responses suggests an important new target for restoring redox equilibrium in spiral ganglion neurons, which are key to MD pathogenesis [11, 17]. For thousands of years, mushrooms have been valued in traditional Asian medicine [18, 19], and they are increasingly emerging in Western nations as essential functional dietary supplements capable of boosting the immune system and counteracting the inflammatory condition [17]. A growing body of research has looked into a variety of mushroom extracts, including *Ganoderma lucidum, Agaricus bisporus, Hericium erinaceus, Pleurotus ostreatus*, and *Coriolus versicolor*, and found that they have a variety of health benefits, including immunomodulatory, antioxidant, anti-inflammatory, antiviral, anticancer, hepatoprotective, and prebiotic properties [20-24]. Furthermore, nutritious mushroom biomass preparations can be utilized as an alternative treatment or in synergistic combination with conventional pharmaceuticals to increase therapeutic activities and lower the risk of negative effects in neuro-degenerative illnesses [5, 25, 26]. *Coriolus versicolor* is emerging as a natural therapeutic candidate due to its significant anti-inflammatory health-promoting properties in animal models, as well as in people [27]. *Coriolus versicolor* is a rich natural source of bioactive chemicals such as minerals, vitamins, carbohydrates, terpenoids, and phenolic acids such as flavonoids, which have a significant potential for preserving human health and preventing many pathological conditions [28-31]. We have been the first to report the presence of specific polyphenols at quite significant concentrations in *Hericium erinaceus* biomass, and this finding has also been confirmed in *Coriolus versicolor* biomass, demonstrating their anti-inflammatory and neuroprotective action associated with the modulation of stress resilience pathways in a rodent model of traumatic brain injury [32, 33]. Recent *in vitro* and *in vivo* studies from our laboratory have provided promising results on the neuroprotective role of mushroom biomass preparations against the neuroinflammatory damage that occurs in Alzheimer's disease [5, 25, 34] and extended the elucidation of their nutritional and biological relevance in a recent clinical study by evaluating the effectiveness of subacute (3 months) treatment with Coriolus biomass preparation in reducing systemic oxidative stress and neuroinflammatory damage in MD patients, where increased levels of vitagene expression associated with an increased plasma GSH/GSSG ratio were found [35] (Fig. **1**). In this study, we extend our previous finding by hypothesizing that chronic supplementation with Coriolus mushroom biomass preparation may have a significant impact on the neurotoxic insult and prolonged pro-inflammatory and oxidative status operating in MD pathogenesis.

## 2 MATERIALS AND METHODS

### 2.1 Chemicals

5,5’-Dithiobis-(2-nitrobenzoic acid) (DTNB), 1,1,3,3-tetraethoxypropane, purified bovine blood SOD, NADH, glutathione (GSH), glutathione disulfide (GSSG), nicotinamide adenine dinucleotide phosphate (β-NADPH, type 1, tetrasodium salt), and glutathione reductase (GR; Type II from Baker’s Yeast), were from Sigma Chemicals Co, St. Louis (USA). Phosphate buffer saline (PBS), NADH, rotenone, decyl ubiquinone, and cytochrome was acquired from Sigma-Aldrich S.r.l. (Milan, Italy). The analytical standards of Prostaglandin F2 α, Prostaglandin E2, Prostaglandin D2, Leukotriene C4, Thromboxane B2, 11-Dehydro Thromboxane B2, 12-(R)-HETE, 9-(S)-Hode, 15-(S)-HEPE, 12-(S)-HEPE, 13-(S)-HODE, 15-(S)-HETE, 8-(S)-HETE, 6-Keto Prostaglandin F1α, 8-Iso Prostaglandin F2α, 10-(S),17-(S)-DIHDHA and deuterated internal standards (IS) were purchased from Cayman chemical (Ann Arbor, USA). Methanol (MeOH), acetonitrile and formic acid 99.9% (LC-MS grade) water (HPLC gradient grade) were supplied from VWR (VWR International PBI S.r.l. Milan, Italy). The IS working solution was prepared by mixing each internal standard at a concentration of 1000 pg/µl with ethanol. The IS working solution and the standards were stored in glass vials at -20℃ for short-term storage. All additional chemicals were from Merck (Darmstadt, Germany) and of the uppermost grade available.

### 2.2 *Coriolus versicolor* Biomass Preparation

*Coriolus versicolor* is found practically everywhere. However, its bioactivity varies depending on the environment in which it grows. To avoid these variances, an established CV-OH1 strain with quick and hostile colonization was employed. As previously described, *Coriolus versicolor* biomass comprising both mycelium and primordia (early fruit body) was obtained using the manufacturer's protocol (Mycology Research Laboratories Ltd. (MRL), Luton, UK) by culturing the biomass on autoclaved substrate. Experiments were conducted using 500 mg tablets of *Coriolus* biomass, including mycelium and primordia of the appropriate fungus, kindly donated by MRL as the commercially available product. The optimal dosage (200 mg/kg) was determined based on the dose used in clinical trials with cancer or human papillomavirus (HPV) patients (3 g/day) [36, 37], which was also verified by rat investigations [34].

### 2.3 Ethical Permission

The study was authorized by the local Ethics Committee (prot. N. 76/2018/PO), and all patients provided informed consent.

### 2.4 Patients

In this study, 50 patients (30 females and 20 males, with an average age of 50.5 +/- 13.6 years; range 30-60 years) with MD have been enrolled according to the diagnostic scale of the Committee on Hearing and Equilibrium of the American Academy of Otolaryngology—Head and Neck Surgery published in 1995 for MD [38]. The diagnostic scale included two or more spontaneous episodes of dizziness of 20 minutes or more, hearing loss on at least one tinnitus, or auditory fullness in the treated ear, documented audiometrically. The patients were divided into two groups, A and B. Group A was made up of 20 patients suffering from cochlear sensorineural hearing loss (SNHL), to whom no treatment was administered, while group B was made up of 30 patients suffering from SNHL, who were treated with a preparation of biomass from the fungus *Coriolus versicolor* (MRL) administered orally in 500 mg tablets (3 tablets every 12 h, morning and evening, for 6 consecutive months). Constituted exclusion criteria: (1) older than 60 years; (2) presence of cardiovascular diseases; (3) presence of metabolic disorders and/or parts; (4) the presence of external ear pathologies and/or medium; (5) the presence of alterations of state-acoustic nerve; (6) prior learning and/or recent treatment with antioxidant drugs or otherwise active in the compartment cochlear. All patients, after anamnestic investigation, underwent the T0 initial phase, where the Profile of Mood States (POMS) questionnaire was administered to assess the emotional and degree of psychological stress status indexed on the basis of specific elements, such as Tension-Anxiety (TA), Depression-Discouragement (D), Anger-Hostility (AH), Vigor-Activity (V), Fatigue (F), Confusion-Loss (C), in relation to the impairment caused in each subject from hearing. The POMS original scale contains 65 self-report items using the 5-point Likert Scale. Participants can choose from 0 (not at all) to 4 (extremely). In addition, all subjects were given a tinnitus questionnaire consisting of 40 multiple-choice questions to define the impact of symptoms on the patient's life. In the groups, the grade of severity for each patient was established on the basis of the vertigo attack frequency over a year (from 2 to 8 crises), the intensity and the duration of symptoms (from a few days to some weeks to a month in the most severe case). In addition, the hearing loss degree was assessed instrumentally, allowing staging of the disease in MD patients. Enrolled patients were also examined to define the qualitative and quantitative characteristics of auditory function: (a) examination ENT, (b) test tone audiometry, (c) speech audiometry, and (d) impedancemetry examination. Such instrumental examinations were aimed at defining not only the extent of hearing loss but also the location of the SNHL to define whether it is cochlear or retrocochlear. In Group B, the mushroom-treated group of patients was accomplished by oral administration of MRL *Coriolus versicolor* biomass compound for 6 consecutive months (T6) to assess its neuroprotective, anti-neuroinflammatory potential and thus test the possible protection against cellular degeneration in general and, particularly, in the inner ear. At 3 months (T3 phase) and then at 6 months (T6 phase) from the beginning of treatment, we evaluated in all patients the degree of an evolutive trend of auditory function as well as oxidative stress, redox status, cellular stress response, and Lipoxin A4, in the blood. Each patient, either in Group A or Group B, at T0, T3 and T6 phases were subjected to blood and urine sampling, and biochemical analysis executed in plasma and lymphocytes to assess Nrf2, stress response protein (vitagenes), glutathione status (reduced glutathione (GSH), oxidized glutathione (GSSG), and GSH/GSSG ratio and lipoxin A4, and in urines for determination of specific markers of lipid and protein oxidation processes. Finally, age-matched healthy subjects (n=36) were chosen as the control group, which included 16 males and 20 females, with an average age of 46.5 +/- 11.4 years, with no cochleo-vestibular disorders or systemic diseases. Criteria of exclusion were determined by audiometric and clinical examinations for those patients showing tympanic membrane perforation, otosclerosis, infectious or neoplastic diseases of the ear and of the acoustic nerve.

### 2.5 Sampling

9 mL of blood was collected by venipuncture from an antecubital vein into three tubes containing ethylenediamine tetraacetic acid as an anticoagulant and were used for lymphocyte purification. All samples were saved at -80℃ until analysis.

### 2.6 Lymphocytes Purification

Lymphocytes were purified from peripheral blood using the Ficoll Paque System according to the manufacturer's instructions (GE Healthcare, Piscataway, NJ, USA).

### 2.7 Determination of Protein

The BCA (bicinchoninic acid) protein assay technique was used to dose proteins [39].

### 2.8 Lipoxin A4 Assay

LipoxinA4 quantity was assessed using an ELISA kit using the company's procedure. A spectrophotometer was used to measure the wavelength of 450 nm.

### 2.9 Western Blot Analysis

Plasma samples were treated to separate lymphocytes, which were then homogenized and utilized for western blot analysis after protein content was determined, as previously reported [35]. Anti-Nrf2, anti-HO-1, anti-Hsp70, anti-GCs, anti-Trx, anti-Sirt-1, anti-HNE, anti-α-synuclein, anti-NF-kB, anti-IKB-α, anti-GFAP, anti-Iba1 polyclonal antibodies (Santa Cruz Biotech. Inc.) were combined in a 5% w/v nonfat dry milk solution and incubated at 4℃ overnight. Blots were then incubated at room temperature for 1 hour with either a peroxidase-conjugated bovine anti-mouse IgG secondary antibody or a peroxidase-conjugated goat anti-rabbit IgG secondary antibody (Jackson Immuno Research, West Grove, PA, USA). To confirm that the protein amounts were comparable, the membranes were further treated with an antibody against β-actin and Lamin A/C (Santa Cruz Biotechnology, Dallas, TX, USA). A Super-Signal West Pico Chemiluminescent Substrate (Pierce) enhanced chemiluminescence detection system reagent was used to detect the signals [40]. The relative expression of the protein bands was measured using densitometry and standardized to β-actin and Lamin A/C levels using Bio-Rad ChemiDoc XRS software [41]. Blot signal images were input into analysis software (Image Quant TL, v2003).

### 2.10 Measurements of Mitochondrial Enzymatic Activities

Centrifugation on a Ficoll-Hypaque density gradient was used to extract peripheral blood cells from K3-EDTA blood, which was then washed twice and frozen at -80℃. The study was authorized by the University of Catania's Institutional Ethical Review Board. PBMCs (o Lymphocyte) pellets were resuspended in PBS and sonicated for 10 seconds at 0℃ to measure spectrophotometric enzymatic activity. The Bio-Rad protein assay was used to assess total protein content. The NADH-UQ oxidoreductase activity (complex I) was measured in 40 mM potassium phosphate buffer, pH 7.4, 5 Mm MgCl_2_, the presence of 3 mM KCN, 1 μg/mL anti-mycin, 200 μM decyl-ubiquinone, and 70 μg of proteins, after the oxidation of 100 μM NADH at 340-425 nm (∆ε = 6.81 mM^−1^). The activity was corrected for the residual activity measured in the presence of 1 µg/mL rotenone [42]. Succinate-cytochrome c oxidoreductase (complex II + III) activity was measured using 50 μg of protein in 25 mM potassium phosphate buffer, pH 7.4, 5 mM MgCl2, 20 mM succinate, 2 mM potassium cyanide (KCN), 65 mM decyl ubiquinone, and 20 mM cytochrome c. The increase in absorbance of cytochrome c was monitored at 550-540 nm (Δε = 19.1 mM^−1^・cm^−1^) 550 [43]. The assay involves oxidized cytochrome c as the electrons acceptor and succinate as donor. The activity of cytochrome c oxidase (complex IV) was determined by monitoring the oxidation of 10 μM ferricytochrome c at 550-540 nm (∆ε= 19.1 mM^−1^ cm^−1^). Enzymatic activity was determined using 50 g of proteins in 10 mM phosphate buffer, pH 7.4 [44].

### 2.11 Lipidomic Analysis

#### 2.11.1 Sample Extraction and Chromatographic Analysis

The urine samples were produced in accordance with previously published techniques [45]. In brief, 50 μl of urine was vortexed with 50 μl of MeOH and 20 μl of IS working solution. The chromatographic separation was performed on a 25℃ XBridge BEH C18 column (21 x 50 mm, 2.5 μm). The mobile phases were H_2_O + 0.1% Formic acid (A) and acetonitrile + 0.1% Formic acid (B), with a flow rate of 0.25 mL/min. The injection volume was 10 μl, and all analytes were eluted between 0.10 and 12 minutes with gradients ranging from 10 to 90% B.

#### 2.11.2 MS Conditions and Validation of the Method

As a mass spectrometer, a Q ExactiveTM Plus Hybrid Quadrupole-OrbitrapTM (Thermo Fisher Scientific, California, USA) with a heated electrospray ionization source was employed (HESI-II). Wolfer *et al*. [46] provided the source parameters. The Full MS scan/dd-MS2 mode was used to collect all data. The resolution of the Orbitrap was adjusted to 140,000 FWHM (scan range 200 to 800 m/z). For a maximum injection period of 100 ms, the automatic gain control (AGC) was set to 1 x 106 ions. The product ions were discovered by raising the normalized collision energy until the precursor ions were completely fragmented. Each analyte was assigned a normalized collision energy (NCE) value. The retention time (tR), accurate mass, and distinctive fragmentation were used to identify the analytes. All analyses were performed with no lock mass. Each day before the study, an external calibration for mass accuracy was done. Thermo Xcalibur TM version 4.0 software was used to record and expound on acquisition data. The method's performance was evaluated for linearity, specificity, and trueness in compliance with Commission Decision 2002/657. The LOQ was computed as the smallest amount of standard required to generate an S/N> 5 while remaining within the calibration curve's linear range (back-calculated residual 20%). The linearity test yielded good results for all analytes tested (r2>0.997). Trueness by recovery yielded values ranging between 80% and 104%.

### 2.12 Statistical Analysis

The results were presented as means SEM of n = 18 trials (MD alone) or n = 22 experiments (MD with *Coriolus*), each of which was done in triplicate unless otherwise specified. Data were examined using one-way ANOVA, and then Duncan's new multiple-range test was used to check all differences. At *p* 0.05, differences were judged significant. One-way ANOVA was utilized to assess the results for western blot analysis, followed by a Bonferroni post hoc test for multiple comparisons. A *p*-value of less than 0.05 was considered significant.

## 3 RESULTS

### 3.1 Auditory Function Assessment

POMS analysis (Table **1**) revealed that in the group treated with *Coriolus*, there was a significant improvement in subjective parameters related to the patient's psycho-emotional status, which was a function of treatment duration when compared to untreated MD patients, where no differences were observed.

Table **2** shows the number of crises, their duration, and the frequency of symptoms among MD subjects prior to and after treatment with Coriolus. A slight but significant improvement in these clinical parameters was observed after 6 months of treatment. In particular, data from the Tinnitus Handicap Inventory (THI) questionnaire (Table **2**), performed to define the clinical grading of tinnitus severity, showed a statistically significant improvement in the group of patients receiving Coriolus, both at 6 months and at 3 months, as compared to the untreated MD group.

**Fig. (2):**
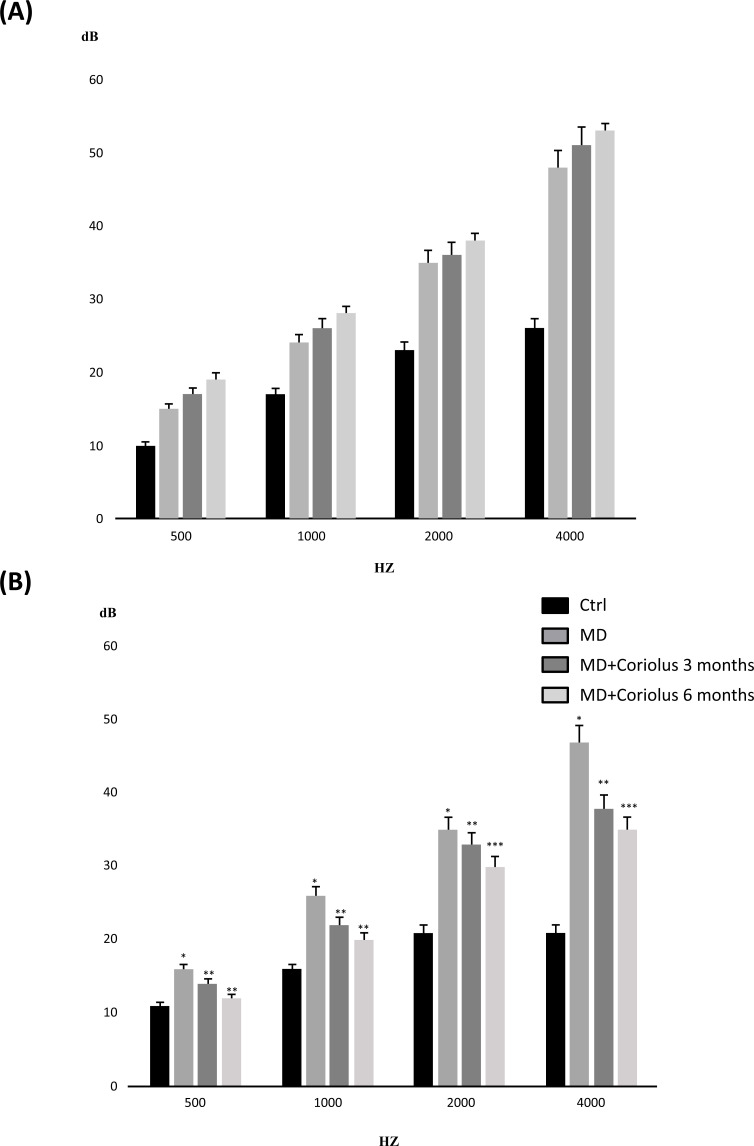
(**A**) Impedenzometric analysis; (**B**) Tonal audiometry analysis. Tonal interest was centered on medium-high frequencies, with an average intensity of 55 db loss. Speech audiometry analysis revealed in subjects receiving mushrooms a significant improvement in intellection threshold, *i.e*., the ability of verbal discrimination, respect to the initial T0 phase, where the threshold of intellection and perception that is 100% of the given words, was found significantly decreased as compared to untreated subjects. *Significant from Control (*p* < 0.05); **Significant from untreated MD subjects (*p* < 0.05); ***Significant from MD plus Coriolus 3 month subjects (*p* < 0.05).

Tonal audiometry was used to assess sensorineural hearing loss in all patients, treated and untreated, at all stages: T0, T3, and T6 (Fig. **2**). In both subject groups, a medium-high frequency with an average strength of 55 dB loss was studied. Tonal audiometry investigation in both untreated patients (MD group) and Coriolus supplementation MD + Coriolus) patients revealed no improvement in medium-high frequency assessed at T3 and T6, compared to beginning phase thresholds (T0). In the same experimental conditions, speech audiometry analysis revealed a significant improvement in intellection threshold, *i.e*., the capacity of verbal discrimination, in the group of subjects having mushrooms compared T0 phase, where the threshold for 100% intellection and perception of the given words was significantly decreased (from 75 dB to 59.2 and 65.1 dB for 6 month and, respectively 3-month treatment). In contrast to the Coriolus biomass-treated groups, we found no significant differences between thresholds assessed at T0 and those measured at later time points in the MD patient group. This finding was consistent with impedance meter measurements at the examination, which revealed in all subjects, either at the T0 initial phase or at the T3 and T6 phases, an average increase in stapedial reflex threshold and positivity of the Metz test, indicating cochlear suffering, with no significant differences between the two groups (data not shown).

### 3.2 Redoxomics: Modulation of Nrf2-dependent Pathway in MD Patients after *Coriolus* Mushroom Supplementation

**Fig. (3):**
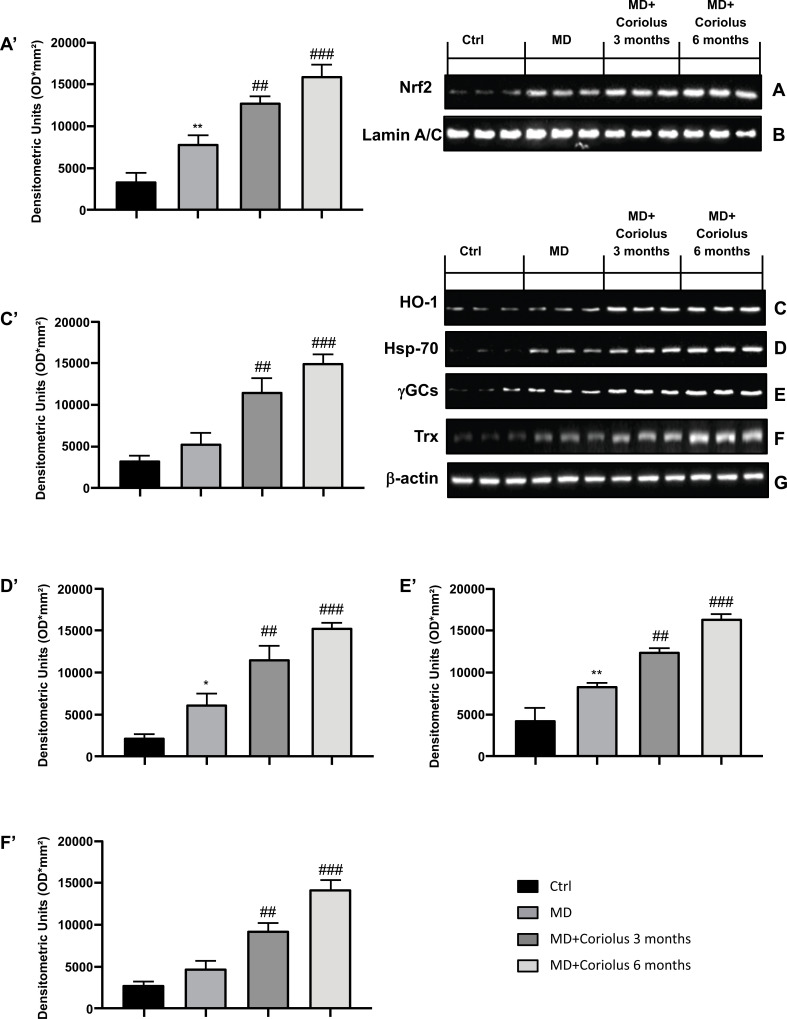
Modulation of Nrf2 pathway. Western blot analysis of Nrf2 expression (**A**) HO-1 (**C**), Hsp-70 (**D**), γGCs (**E**) and Trx (**F**). The expression of the protein bands was measured using densitometry (**A’**, **C**,**’** **D**,**’** **E’** and **F’**) and standardized to β-actin (**G**) and Lamin A/C levels (**B**). *p<0.05 *versus* Ctrl; **p<0.01 *versus* Ctrl; ##*p*<0.05 *versus* MD; ###*p*<0.001 *versus* MD.

ROS can oxidize proteins and membrane lipids, generating carbonyls and lipid hydroperoxides, as well as many aldehydes such as HNE, which can accumulate in cells at a relatively high concentration and cause cell toxicity. Oxidative stress interacts with the Nrf2 pathway. This transcription factor is in the cytoplasm, bound to Keap1. Activation of Nrf2 pathway leads to Nrf2 translocation into the nucleus by transactivating the antioxidant response elements (AREs) of several cytoprotective genes, resulting in upregulation of Nrf2-dependent vitagenes. Fig. (**3A** and **3A’**) show Nrf2 expression in lymphocytes, which is maximally expressed in subjects supplemented with *Coriolus* for 6 months, compared to 3 months of treatment. However, Nrf2 expression in subjects receiving mushroom biomass was significantly higher than in MD patients not receiving any supplementation, whereas minimal levels of expression of Nrf2 were found in normal healthy controls. In conditions promoting proteotoxicity, such as those occurring in MD pathophysiology, cells respond adaptively with increased synthesis and accumulation of several members of stress proteins, primarily Hsp70 and the Nrf2-regulated HO-1. Consistent with our previously published results, mushroom supplementation with *Coriolus* biomass resulted in up-regulation of the inducible isoforms of both Hsp70, heme oxygenase-1 (HO-1), as well as γ-GC ligase and thioredoxin, determined in lymphocytes (Fig. **3A**-**F**), as compared to untreated group of MD patients or healthy controls. Representative Western blots obtained probing tissue samples with specific antibodies are shown in Fig. (**3A’**-**F’**), respectively. Expression of these proteins was found at the highest levels in MD receiving *Coriolus* for 6 months, with respect to subjects treated for 3 months, as compared to the MD group in which these proteins were, however, significantly higher than the levels found in healthy volunteers taken as control.

Analyzing sirtuin-1 expression yielded similar findings. Sirtuin-1 immunoreactivity was greater in lymphocytes of MD patients treated for 6 months and 3 months with *Coriolus*, as shown in Fig. (**4A**, **A’**), than in the untreated MD group, where we observed expression levels equal to those seen in healthy controls.

It is well established that oxidative stress and altered thiol status correlate with systemic redox imbalance and oxidative stress, as found in various neuroinflammatory brain diseases. Hence, the content of total GSH, reduced and oxidized glutathione and the GSH/GSSG ratio was determined in the plasma of MD patients as a measure of the antioxidant status and compared with the levels of *Coriolus*-treated MD group (Table **3**). We report in the plasma from MD patients significantly lower levels of both total glutathione and GSH, as compared to controls. In the group of *Coriolus*-supplemented patients, significantly higher levels of either total glutathione or GSH were measured in comparison to the MD group alone, with a content that was comparable to normal control levels. This finding paralleled corresponding significantly higher GSSG levels (*p* < 0.05) found in MD patients with respect to normal controls or *Coriolus-*supplemented MD patients, where no statistically significant differences were found between 6 months of treatment with *Coriolus* and healthy controls (Table **3**). These changes resulted in a plasma GSH/GSSG ratio that was significantly lower in MD subjects compared to healthy controls, while *Coriolus* supplementation resulted in a higher GSH/GSSG ratio than in untreated MD patients, with levels comparable to non-pathological normal values measured in MD patients receiving *Coriolus* for 6 months.

### 3.3 Assessment of Systemic Oxidative Status

**Fig. (5):**
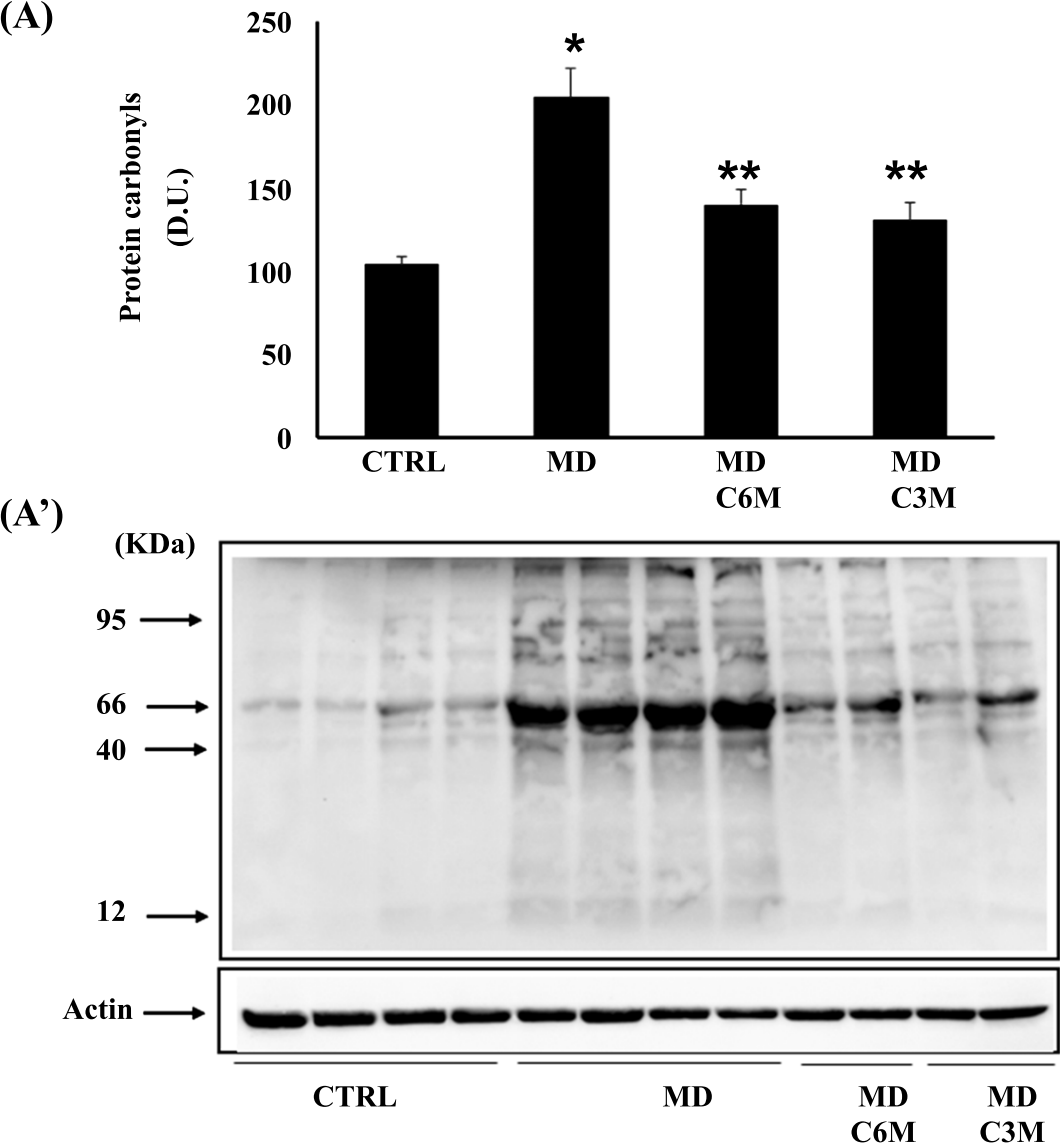
Effect of *Coriolus* on protein carbonyls in MD patients. (**A**) Densitometric evaluation: the bar graph shows the values are expressed as mean standard error of mean of 3 independent analyses. *P* < 0.05 *vs* control. (**A’**) A representative immunoblot is shown. β-actin has been used as loading control. D.U., densitometric units. *Significantly different from control (*p* < 0.05), **Significantly different from MD alone (*p* < 0.05).

**Fig. (6):**
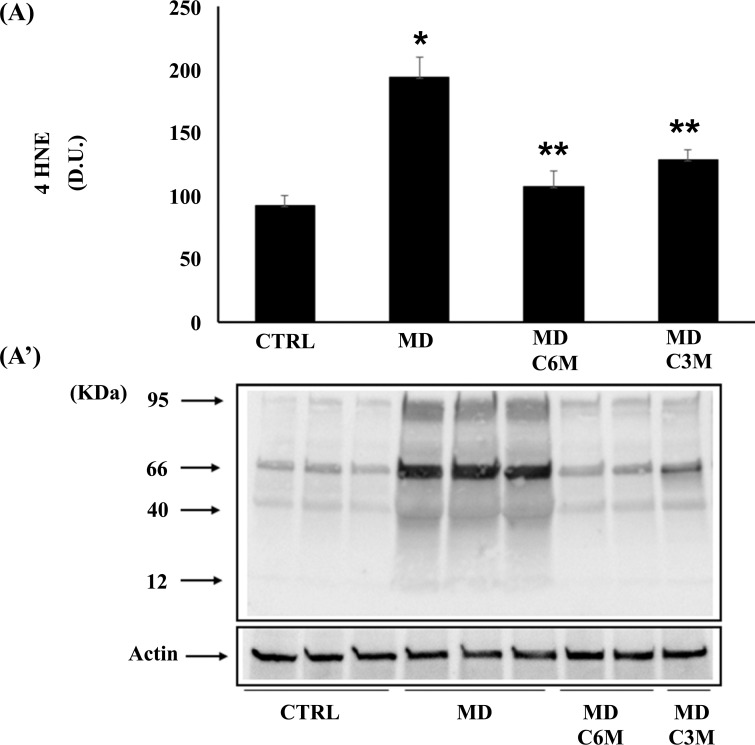
Effect of *Coriolus* on 4 HNE in MD patients. (**A**) Densitometric evaluation: the bar graph shows the values are expressed as mean standard error of mean of 3 independent analyses. *P* < 0.05 *vs* control. (**A’**) A representative immunoblot is shown. β-actin has been used as loading control. D.U., densitometric units. *Significantly different from control (*p* < 0.05), **Significantly different from MD alone (*p* < 0.05).

Being generally unrepairable and leading to the production of potentially harmful protein aggregates and to cellular dysfunction, oxidative damage to proteins produced by the free-radical attack by binding *via* Michael addition to proteins, particularly at cysteine, histidine, or lysine residues, promotes protein carbonylation and thus exerts deleterious effects on cell function and viability. Under conditions of oxidative stress, lipid oxidation products, measured by HNE and spontaneous ultraweak chemiluminescence, also accumulate [9]. Protein and lipid oxidation occurring because of oxidative stress generates the formation of carbonyl groups in amino acid residues and, respectively, of 4-hydroxynonenal (HNE) from arachidonic acid or other unsaturated fatty acids. Protein carbonyls (Fig. **5A**) and HNE (Fig. **6A**) were examined in lymphocytes of MD patients in the absence or in the presence of supplementation with *Coriolus* and compared to controls. In lymphocytes of MD patients, protein carbonyls, measured as DPNH-reactive material, resulted significantly higher in MD patients than in healthy controls. After supplementation with *Coriolus* biomass, we found a significant reduction of protein oxidation both at 6 and 3 months. Fig. (**6A**) reports lymphocytes levels of HNE, which were higher in MD patients as compared to normal controls, whereas supplementation with *Coriolus*, resulted in a decreased HNE formation, which was at 6 months higher than 3 months. Representative blots are reported in Figs. (**5A’** and **6A’**), respectively. Healthy consistently, Fig. (**7**) shows spontaneous ultraweak chemiluminescence (UCL) in lymphocytes, which is significantly increased in MD patients, compared to healthy controls, and the effect of mushroom supplementation, which induces a significant time-dependent decrease of oxidation products.

### 3.4 Effects of *Coriolus Versicolor* on Mitochondrial Respiratory Complex Activities

In neuroinflammatory conditions, oxidative stress is often coupled with mitochondrial dysfunction, resulting in a vicious cycle that promotes neurodegeneration and impairs brain cell function. As a result, we investigated mitochondrial complex activities in lymphocytes from MD patients in the absence or presence of *Coriolus* mushroom biomass treatment. Fig. (**8**) reports complex I, complex II-III, and complex IV respiratory chain activity in our various patient groups. Interestingly, we found a significant decrease in NADH dehydrogenase (Complex I) and Cytochrome oxidase (Complex IV) activity in MD patients' lymphocytes when compared to normal controls. In contrast, mushroom treatment for 6 months restored both mitochondrial complex activities to near-control levels.

Under the same experimental settings, we discovered a considerable increase in Complex II-III activity in MD participants when compared to control values, but mushroom treatment (6 months) returned this complex to normal levels, as seen in control subjects.

### 3.5 Effects of *Coriolus Versicolor* on Inflammatory Processes

**Fig. (9):**
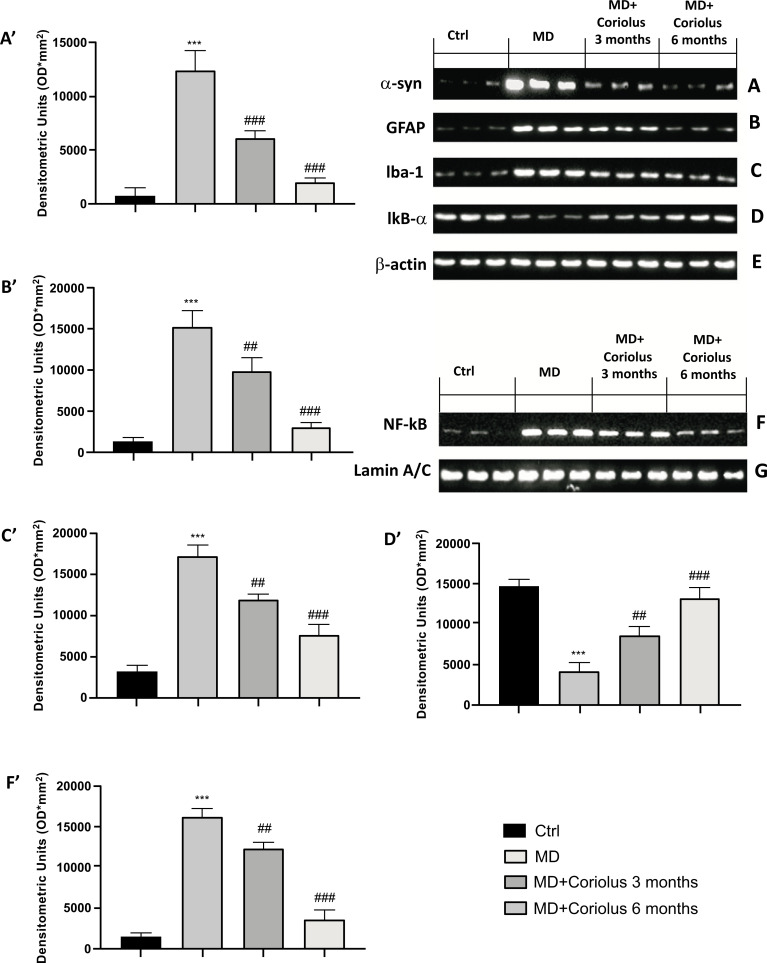
Effects of *Coriolus* on α-syn, GFAP, Iba-1and NF-kB pathway. Western blot analysis of α-syn expression (**A**) GFAP (**B**), Iba-1 (**C**), IKB-α (**D**) and NF-kB (**F**). The expression of the protein bands was measured using densitometry (**A’**, **B’**, **C’**, **D’**, and **F’**) and standardized to β-actin (**E**) and Lamin A/C levels (**G**). ***p < 0.001 *versus* Ctrl; ##*p* < 0.05 *versus* MD; ###*p* < 0.001 *versus* MD.

In addition to oxidative stress, we wanted to analyze the expression of neuroinflammatory protein patterns, such as α-synuclein, as well as GFAP, Iba-1 and the NF-kB pathway involved in the maturation of immune cells and, respectively, in the inflammatory burden. As expected, the results obtained showed an increase in α-synuclein, GFAP and Iba-1 proteins in the MD group, while *Coriolus* treatment at 3 months but even more at 6 months was able to reduce the expression of these proteins (Fig. **9A**-**C** and **A'**-**C'**). Furthermore, we observed how *Coriolus* treatment was able to act on the NF-kB pathway. Specifically, patients with Meniere's had increased NF-kB levels against a decrease in its inhibitor IKB-α. An opposite trend was observed in patients treated with *Coriolus* for 3 and 6 months, with better action at 6 months (Fig. **9D**-**F** and **D'**-**F'**).

### 3.6 Lipidomics Analysis

**Fig. (10):**
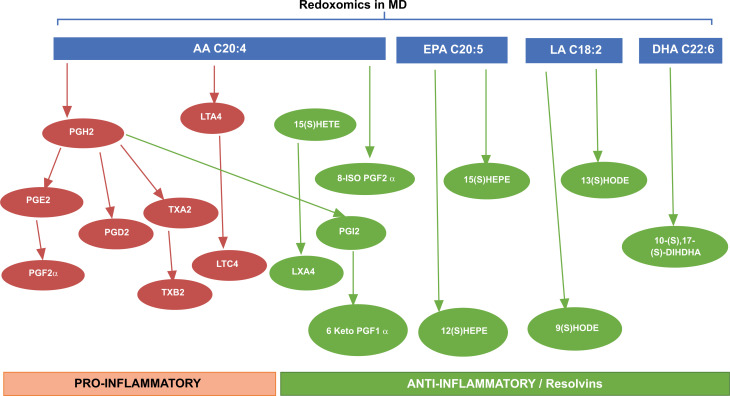
Lipidomics in MD patients in the presence and absence of supplementation with nutritional mushrooms. Scheme illustrating the development of mass spectrometry platforms enabling quantification of diverse oxylipin species in human urine as a tool to understand metabolic redox homeostasis in normal as well as MD pathophysiological conditions.

Quantification of diverse lipid species in human urine is of considerable importance in normal and pathophysiological conditions to study redox metabolic homeostasis, and this identification is possible by mass spectrometry platforms. Oxidation of polyunsaturated fatty acids allows the synthesis of bioactive lipids: linoleic acid, dihomo-γ-linolenic acid, arachidonic acid, eicosapentaenoic acid, and docosahexaenoic acid generate bioactive lipids. The development of mass spectrometry platforms enabling quantification of diverse lipid species in human urine is of crucial importance to understand metabolic redox homeostasis in normal, as well as pathophysiological conditions (Fig. **10**).

Here, we clearly demonstrate that administration of *Coriolus* to MD patients significantly increases LXA4, a bioactive eicosanoid endowed with high anti-inflammatory potential, as measured in plasma, lymphocytes, and urines of MD patients prior to and after mushroom treatment, in comparison to the control group. These results are reported in Fig. (**11A**-**C**).

**Fig. (12):**
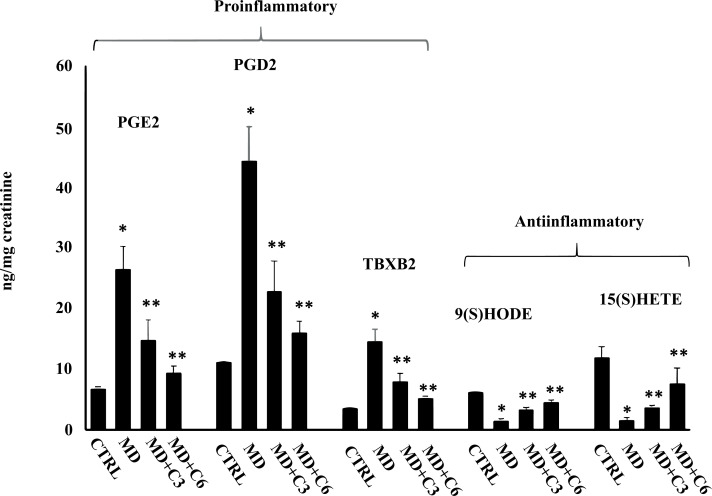
Effect of *Coriolus* administration on proinflammatory and anti-inflammatory eicosanoids measured in urines of MD patients. *Significantly different from control (*p* < 0.05), **Significantly different from MD alone (*p* < 0.05).

**Fig. (13):**
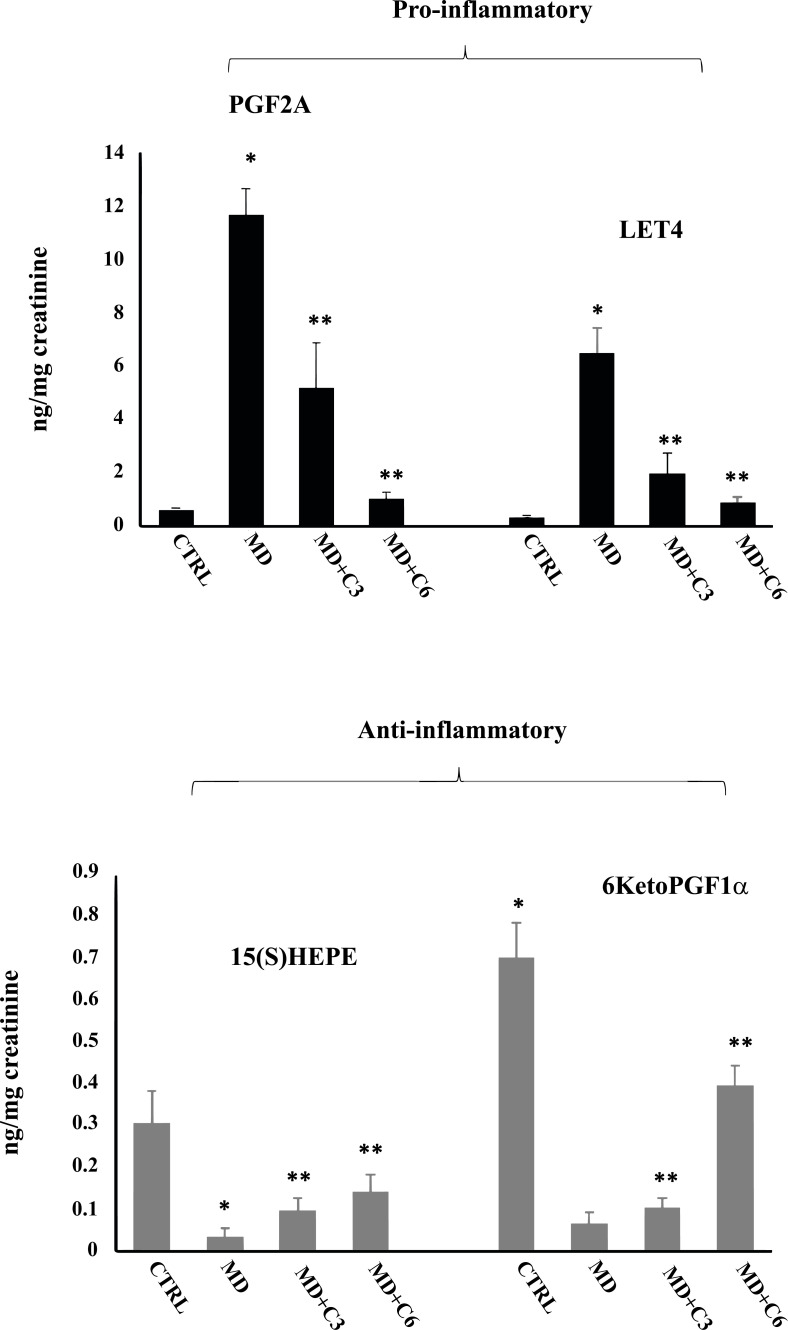
Effect of *Coriolus* administration on proinflammatory and anti-inflammatory eicosanoids measured in urines of MD patients.

Additionally, a panel of anti-inflammatory eicosanoids was measured, and the results are shown in Figs. (**12** and **13**). 9(S)HODE, 15(S)HETE, 15(S)HEPE and 6KetoPGF1α were significantly reduced in the urine of MD patients compared to controls, while mushroom supplementation increased considerably urinary levels of this inflammatory component, an effect that at 6 months was greater than at 3 months. Consistently, analysis of urine levels of pro-inflammatory eicosanoids isoprostane PGF2α, PGE2, PGD2, 11-dehydro TXB2 and LTC4 showed the opposite results with significant increases in MD patients compared to controls, while after mushroom supplementation, a significant reduction of all measured proinflammatory eicosanoids levels was found at 6 months significantly greater than at 3 months (Figs. **12** and **13**). Taken together, our finding is consistent with the notion that altered thiol status and oxidative stress in degenerating neurosensorial organs correlate with systemic oxidative stress and redox imbalance.

## 4 DISCUSSION

MD is a chronic multifactorial disorder induced by the neurodegeneration of the hair cells of the spiral ganglion in the inner ear that results in a series of symptoms, including tinnitus, fluctuating deafness, vertigo and hearing loss that negatively affect the quality of life of the subjects [46-51]. Numerous causal factors have been considered in the development of cochleovestibular dysfunctions, but the origin of MD is still undefined [52]. For example, endolymphatic hydrops is recognized as an important histopathological hallmark of MD, which, according to brain nuclear medicine imaging of the brain (SPECT) (single photon emission computed tomography), appears to be due to interferences in homeostasis in the blood-brain labyrinth. However, experimental data from animal models for this disorder have failed to identify the mechanism by which endolymphatic hydrops cause hearing loss [53, 54]. Recent evidence highlights the crucial role of neuroinflammation and oxidative stress in the development of endolymphatic hydrops, which can lead to apoptosis of spiral ganglion neurons and contribute to sensorineural hearing loss (SNHL) that occurs in advanced stages of MD [48]. Spiral ganglion cells and hair cells in both human and animal studies have shown severe deterioration with detailed ultrastructural changes of a significant decrease in dendritic innervation densities, explaining the associated severe sensorineural hearing loss [55, 56]. In advanced stages of MD, increased neuroinflammation and oxidative stress promote apoptotic death of spiral ganglion neurons [48, 57]. Mushrooms, which have been used in traditional medicine for several years [30, 58], have gained importance as nutritional components of a diet capable of modulating inflammation and immunity. Many studies have examined many biological actions associated with mushroom extracts, including anticancer, immunomodulatory, antioxidant, antiviral, antibacterial, and hepatoprotective effects [58,59]. Medicinal effects have been demonstrated for many traditionally used mushrooms, including extracts of *Pleurotus ostreatus, Agaricus campestris* and *Coriolus versicolor* [60]. The active ingredient derived from *Coriolus versicolor* belongs to a new category of substances known as biological response modifiers (BRM), which can enhance the immune system and provide various therapeutic benefits. Recent literature has documented the therapeutic value of medicinal mushrooms, such as *Coriolus versicolor*, against various chronic inflammatory disorders, including neurodegenerative, cardiovascular and metabolic diseases, as well as viral infections and cancer. Using analyses of Liquid Chromatography-Orbitrap-Mass Spectrometry (LC-Orbitrap-MS) and Gas Chromatography-Tandem Mass Spectrometry (GC-MS/MS), our group was the first to identify the active polyphenols of *Coriolus versicolor* and *Hericium herinaceus*, providing strong evidence for a novel neuroprotective strategy to reduce inflammation, oxidative stress, and neurodegenerative insult, in a process associated with the upregulation of cytoprotective proteins, including vitagenes in a rat model of traumatic brain injury [32]. Consequently, we showed a significant abundance of kaempferol (6.24%), quercetin (6.95), epicatechin (7%), apigenin (9.34%), ferulic acid (10.03%), vanillic acid (11.93%), as the main polyphenols present in *Coriolus* and *Hericium* biomass [32]. Furthermore, cells have evolved response networks to respond to environmental changes and resist various types of injury by sensing and regulating different forms of stress [29, 61]. Viewing MD as an established model of cochlear ganglion neurodegeneration, in our previous studies, we focused on MD neuropathology as a model of measurable neurodegenerative injury, demonstrating alterations in redox status and lipid signatures, together with changes in stress response mechanisms in the cochlear ganglion. MD patients could be improved by subacute administration of redox-active substances, including the nutritional mushroom *Coriolus versicolor* [62, 63] . In this study, we first confirmed our previous data on cellular stress markers in MD patients responding to 3-month treatment with *Coriolus* as a source of polyphenolic antioxidant supplementation. Furthermore, we wanted to evaluate how this fungus acts on defense systems antioxidants even after 6 months of treatment. We investigated oxidative stress markers in MD patients and their changes during *Coriolus* treatments. MD subjects, compared to healthy controls, showed upregulated levels of Nrf2 and Nrf2-dependent proteins (vitagenes) such as HO-1 and γ-GC lyase, as well as Hsp70, Trx and sirtuin-1, associated with increased oxidative stress, as shown by HNE, protein carbonyls and ultra-weak chemiluminescence, measured in lymphocytes, which was associated with a significant decrease in the plasma GSH/GSSG ratio [64]. Chronic inflammation supports the progression of the neurodegenerative insult in particular neuronal districts, such as the spiral ganglion cells in the cochleovestibular apparatus, which also includes the blood-labyrinth barrier, more integrally as a neurobiological unit [65]. However, finding mechanisms to address the pro-inflammatory environment caused by MD pathology is still an ongoing research topic. Lipid mediators have a key role in the perpetuation of inflammatory events. Urine for lipidomics analysis produces a large number of different lipid mediators, including eicosanoids, octadecanoids, and docosanoids produced by cyclooxygenase, lipoxygenase, and cytochrome P450 activities [61, 66, 67]. Lipidomics analysis conducted on MD patients showed upregulation of eicosanoids in urine levels, including F2-isoprostanes and lipoxin A4. However, MD subjects supplemented with *Coriolus* showed a significant decrease in oxidative stress and proinflammatory eicosanoids in association with an increase in anti-inflammatory eicosanoids, including lipoxin A4, associated with the upregulation of stress-sensitive vitagenic proteins. These changes were greater in subjects chronically supplemented for 6 months than in patients treated for 3 months. LXA4 signaling could be a potential therapeutic target for MD-related cochleovestibular dysfunctions and inflammation. LXA4 inhibits neutrophil activation and recruitment, blocks the generation of pro-inflammatory cytokines and toxic compounds, including ROS, supports resolution of the inflammatory process, and acts as an endogenous 'break signal' of inflammation [68]. For this reason, we wanted to evaluate for the first time whether this cascade of events also acted on the NF-kB pathway, which appears to play such an important role in this pathology that it could serve as a biomarker to distinguish between patients with MD and vestibular migraine [69-71]. In this regard, we observed an increase of NF-kB levels and a decrease of IKB-α in MD patients, while *Coriolus* treatment at 3 and 6 months, with greater action of longer treatment, managed to re-establish normal levels expression of these proteins. Furthermore, again, for the first time, we observed the expression of two proteins responsible for inflammatory processes such as GFAP and Iba-1. Our results showed that GFAP and Iba1 levels in MD patients were significantly increased, while a reduction was observed in patients treated with *Coriolus,* especially for 6 months. Another important fact that we have analyzed and that has been studied for the first time in this pathology is the expression of α-synuclein. Several studies have shown that this protein has an important role in lymphocyte maturation and immune cell function [72, 73]. Therefore, the increase in α-synuclein could be responsible for the increase of these cells in MD patients [74, 75]. In our previous studies, we have already demonstrated the neuroprotective potential of *Coriolus* biomass in a rotenone-induced PD model in mice, where the effect was associated to a decreased expression of α-synuclein protein, thus resulting in a limitation of disease progression [76]. The results obtained in this study confirm previous findings, demonstrating a significant increase of α-synuclein expression in MD patients and a significant reduction of this protein in patients treated with *Coriolus* for 3 months and even more for 6 months. Therefore, molecules capable of reducing a-synuclein and, consequently, the inflammatory and oxidative processes underlying MD could represent an innovative strategy relevant to MD physiopathology and therapy. Of course, further studies are needed to understand the real mechanism of action and whether a-synuclein could act as a new therapeutic target.

## CONCLUSION

Our data based on a nutritional approach with *Coriolus* biomass supplementation represent a promising and innovative strategy against inflammation to reduce the aftermaths correlated with oxidative stress-induced neurodegenerative damage of cochleovestibular diseases, including MD but also all pathology associated with sensorineural hearing loss (SSNHL) [67]. Although there are few studies on the characterization of mushroom-derived active ingredients, which makes them very difficult to merge with pharmaceutical practices that prefer purified compounds and, therefore, problematic to patent as complex mixtures, a better understanding of the neurobiological potential of these same complex nutritional mixtures, may pave the way for novel approaches in healthy aging medicine and antidegenerative therapeutics.

## Figures and Tables

**Fig. (4):**
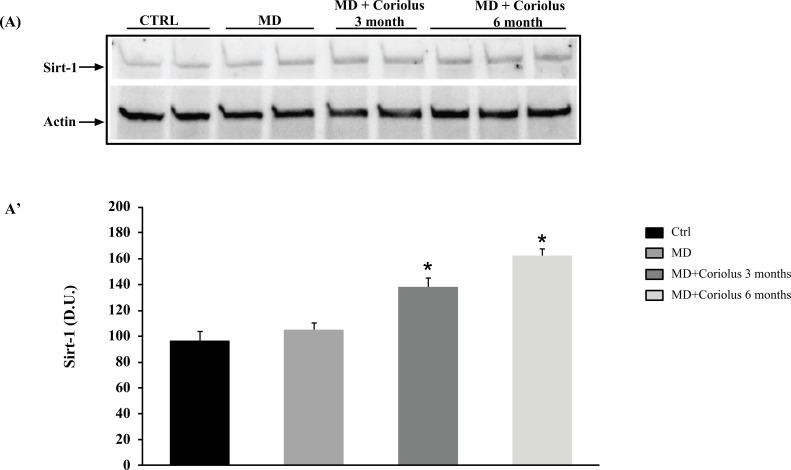
Modulation of Situin-1 by *Coriolus*. Western blot analysis of Sirt-1 expression (**A**) and densitometric analysis (**A’**). *Significantly different from MD alone (*p* < 0.05).

**Fig. (7):**
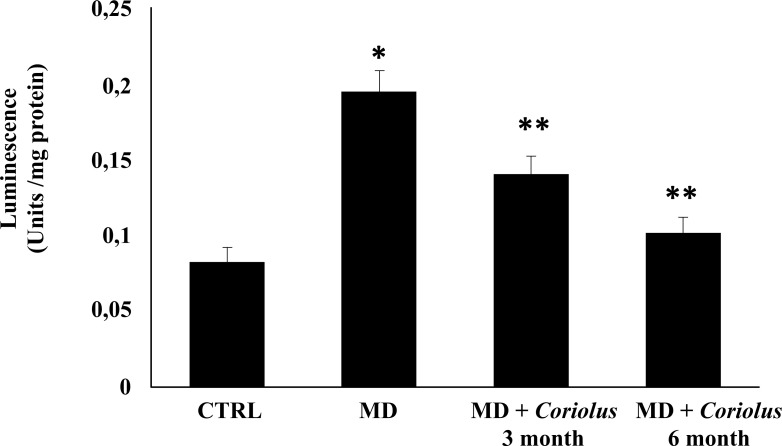
Ultraweak chemiluminescence in lymphocytes. *Significantly different from control (*p* < 0.05), **Significantly different from MD alone (*p* < 0.05).

**Fig. (8):**
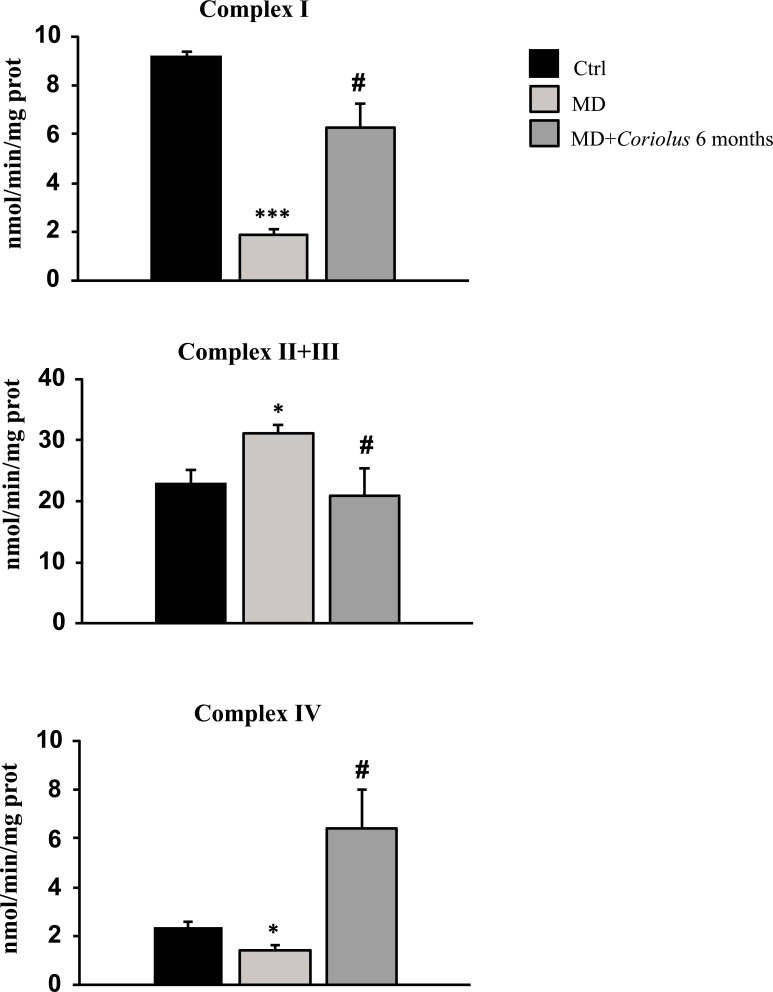
Effect of *Coriolus* on respiratory chain activity of complex I, complex II-III and complex IV in MD patients. *Significantly different from control (*p* < 0.05); *Significantly different from control (*p* < 0.001); # Significantly different from MD alone (*p* < 0.001)

**Fig. (11):**
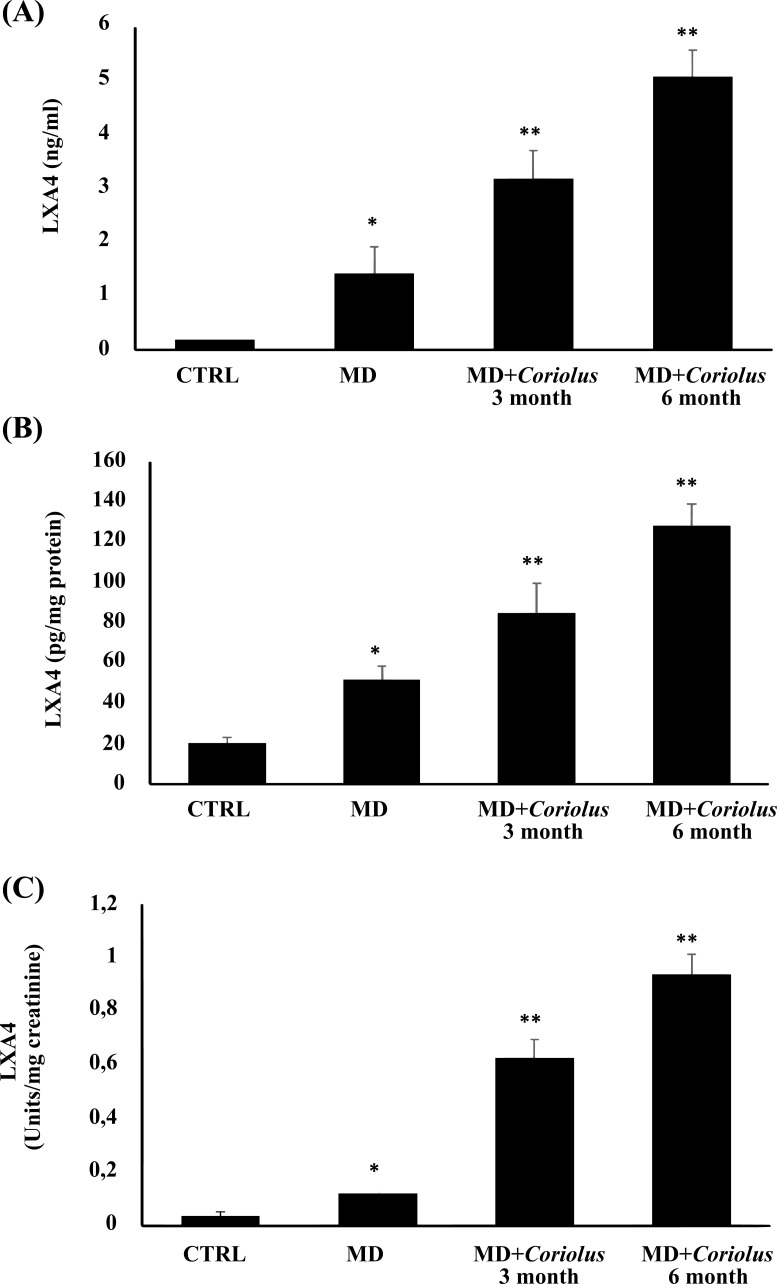
Role of *Coriolus* administration on LXA4 measured in plasma (**A**), lymphocytes (**B**), and urines (**C**) of MD patients. *Significantly different from control (*p* < 0.05), **Significantly different from MD alone (*p* < 0.05)..

**Table 1:** Profile of mood states (POMS).

**Group A**	**Score**		
**MD**			
**Mood of Profile**	**T0**	**T3**	**T6**
Anger	30 ± 2.5	33 ± 3.1	31 ± 2.9
Confusion	18 ± 2.2	20 ± 2.9	19 ± 2.7
Depression	42 ± 3.1	40 ± 2.5	43 ± 2.9
Fatigue	18 ± 2.1	17 ± 2.2	20 ± 2.9
Tension	32 ± 3.1	32 ± 2.7	33 ± 2.7
Vigour	20 ± 2.3	22 ± 2.5	21 ± 2.5
**Total Mood Disturbance (0 to 200)**	160 ± 15.5	164 ± 16.1	167 ± 16.7
**Group B**	**Score**		
**MD + *Coriolus***			
**Mood of Profile**	**T0**	**T3**	**T6**
Anger	32 ± 2.7	20 ± 1.5	15 ± 1.5
Confusion	21 ± 3.1	13 ± 1.8	10 ± 1.8
Depression	42 ± 2.6	28 ± 1.8	15 ± 1.8
Fatigue	16 ± 2.9	14 ± 1.5	10 ± 1.5
Tension	31 ± 2.7	18 ± 2.1	16 ± 1.8
Vigour	20 ± 2.1	15 ± 1.8	12 ± 1.5
*Total mood disturbance (0 to 200)*	162 ± 16.3	108 ± 10.8*	78 ± 10.3**

**Note:** *Significant from untreated MD subjects, **Significant from MD subjects treated for 3 months.

**Table 2:** Questionnaire for assessing the frequency and duration of crisis and symptoms.

**Crisis Frequency**	**Group A**	**MD**	
**Vertigo Attack Frequency**	**T0**	**T3**	**T6**
<2 crisis/6-month	3 (13.6%)	4 (18.2%)	5 (22.7%)
From 3 to 5 crisis/6-month	10 (45.5%)	11 (50.0%)	12 (54.5%)
From 6 to 8 crisis/6-month	7 (31.8%)	5 (22.7%)	3 (13.6%)
**Crisis Duration**			
<1 h	14 (63.6%)	15 (68.2%)	14 (63.6%)
>24 h	6 (27.3%)	5 (22.7%)	7 (31.8%)
**Duration of Symptoms**			
A few days	15 (68.2%)	16 (72,7)	16 (72,7)
Some weeks	4 (18.2%)	2 (9.1%)	3 (13.6.%)
A month	1 (4.5%)	2 (9.1%)	1 (4.5%)
**Tinnitus Handicap Inventory**	75±2.46	76±2.67	76±2.67
**Crisis Frequency**	**Group B**	**MD + Coriolus**	
**Vertigo Attack Frequency**	**T0**	**T3**	**T6**
<2 crisis/6-month	6 (21.4%)	4 (14.3%)	3 (10.7%) *
From 3 to 5 crisis/6-month	12 (42.9.4%)	10 (35.7%)	8 (28.6%) *
From 6 to 8 crisis/6-month	9 (32.1%)	8 (28.6%)	7 (25.0%)
**Crisis Duration**			
<1 h	14 (50.0%)	12 (42.9%)	10 (35.7%) *
>24 h	8 (28.6%)	6 (21.4%)	5 (17.9%)
**Duration of Symptoms**			
A few days	17 (60,7)	15 (53.6%)	12 (42.9%) *
Some weeks	8 (28.6%)	5 (17.9%)	5 (17.9%)
A month	2 (7.1%)	1 (3.6%)	1 (3.6%)
**Tinnitus Handicap Inventory**	76±2.67	52±1.98*	50±1.72*

**Note:** *Significant from untreated MD subjects (*p* < 0.05).

**Table 3:** Total GSH, reduced (GSH) and oxidized (GSSG) glutathione levels and GSH/GSSG ratio in control and MD patients treated with *Coriolus*.

-	**Control**	**Plasma (nmol/ml)**		
		**MD**	**MD+ Coriolus 3 Months**	**MD+ Coriolus 6 Months**
**Total GSH**	17.2 ± 2.2	7.83 ± 3.1*	13.03 ± 2.3**	14.9 ± 2,8**
**GSH**	16.42 ± 2.1	7.14 ± 1.9*	12.04 ± 1.4**	14.08 ± 1.6**
**GSSG**	0.129 ± 0.01	0.172 ± 0.01*	0.151 ± 0.01**	0.104 ± 0.02**
**Ratio GSH/GSSG**	127.3 ± 11	41.5 ± 8.5*	79.03 ± 10**	135.3 ± 12**

**Note:** *Significantly different from control (*p* < 0.05), **Significantly different from MD alone (*p* < 0.05).

## Data Availability

The data that support the findings of this study are available in the methods of this article. The rest of the data will be available from the corresponding author upon reasonable request.

## LIST OF ABBREVIATIONS

AGC: Automatic Gain Control
AH: Anger-Hostility
ALX: LXA4 Receptor
AREs: Antioxidant Response Elements
BCA: Bicinchoninic Acid
BLB: Blood-labyrinthic Barrier
C: Confusion-Loss
D: Depression-Discouragement
DTNB: 5,5’-Dithiobis-(2-nitrobenzoic acid)
F: Fatigue
FPRL1: Formyl-peptide Receptor Like 1
GC-MS/MS: Gas Chromatography-tandem Mass Spectrometry
GSH: Glutathione
GSSG: Glutathione Disulfide
HESI-II: Heated Electrospray Ionization Source was Employed
HNE: 4-Hydroxynonenal
HO-1: Heme Oxygenase-1
HPV: Human Papilloma Virus
KCN: Potassium Cyanide
LC-Orbitrap-MS: Liquid Chromatography-orbitrap-mass Spectrometry
MD: Meniere's Disease
MeOH: Methanol
MRLs: Mushroom
NADPH: Nicotinamide Adenine Dinucleotide Phosphate
NCE: Normalized Collision Energy
PBS: Phosphate Buffer Saline
POMS: Profile of Mood States
SNHL: Sensorineural Hearing Loss
SV: Stria Vascularis
TA: Tension-Anxiety
THI: Tinnitus Handicap Inventory
tR: Retention Time
UCL: Ultraweak Chemiluminescence
V: Vigor-activity
